# Supplementary material for: New evidence for the co-occurrence of two genera of Paleoparadoxiidae (Mammalia, Desmostylia) from the Middle Miocene of Japan: insights into taxonomic status and paleodiversity in Desmostylia
Source: PeerJ. 2025 Jul 31;13:e19578. doi: 10.7717/peerj.19578 (PMC12318504; doi:10.7717/peerj.19578)
Supplement: Supplemental Information 1 [file peerj-13-19578-s001.docx]

# **List of valid taxa with chronostratigraphic assessment**

## ***Seuku emlongi Domning et al., 1986***

**Age range:** 30.60-28.00 Ma.

**Occurrence:** East Pacific, Yaquina Formation, Oregon, USA.

**Stratigraphic distribution:** *Domning et al. (1986)* and *Beatty & Cockburn (2015)* considered the Yaquina Formation as the Late Oligocene (Zemorrian). *Prothero et al. (2001b)* estimate the age of this Formation to be 30.60-28.00 Ma based on the magnetostratigraphy.

## ***Behemotops proteus Domning et al., 1986***

**Age range:** 30.60-23.70 Ma.

**Occurrence:** East Pacific, Sook Formation, Vancouver Island, Canada and Pysht Formation, Washington, USA.

**Stratigraphic distribution:** The Pysht Formation, *Prothero et al. (2001a)* gives age of 30.50-23.70 Ma based on magnetostratigraphy, but *Shipps et al. (2019)* assigned the more precise age of 30.60-28.30 Ma. The Sooke Formation is temporally comparable to the Pysht Formation with an age of 30.50-23.70 Ma (*Brandon et al., 1998; Prothero et al., 2008*).

## ***Behemotops katsuiei Inuzuka, 2000***

**Age range:** 27.40-23.80 Ma.

**Occurrence:** West Pacific, Morawan Formation, Hokkaido, Japan.

**Stratigraphic distribution:** Known only from upper tuffaceous siltstone of the Morawan Formation. The age is based on potassium-argon (K-Ar) dating and fission-track (FT) dating (*Matsui & Ganzawa, 1987*), which suggest a late Oligocene age of 27.4 ± 1.5 Ma (K-Ar)-23.8 ± 2.0 Ma (FT).

## ***Archaeoparadoxia weltoni Clark, 1991***

**Age range:** 24.00-20.40 Ma.

**Occurrence:** East Pacific, Skooner Gulch Formation, California, USA.

**Stratigraphic distribution:** According to *Clark (1991),* the age of this formation is approximately 24.00 Ma. However, other studies have assigned it to the Aquitanian stage (23.00-20.40 Ma) based on biostratigraphy (*Philips et al., 1976; Barboza et al., 2017*).

## ***Paleoparadoxia tabatai Tokunaga, 1939***

**Age range:** 23.80-9.00 Ma.

**Occurrence:** Fossil occurrences of *P. tabatai* range from the USA, Mexico and Japan (*e.g., Reinhart, 1959; Inuzuka, 2005*).

**Stratigraphic distribution:** The oldest record of *Paleoparadoxia* comes from Sankebetsu Formation, dated to 23.8±1.5-20.6±1.0 Ma based on fission-track (FT) dating (*Matsui & Kawabe, 2016*). The youngest record comes from Santa Margarita Formation assigned Tortonian (*Reinhart, 1959*), but numerical age is still debated (11.6-10 Ma?). Our study follows *Matsui & Tsuihiji (2019)* in assigning a minimum age of approximately 9.00 Ma.

## ***Neoparadoxia repenningi Domning & Barnes, 2007***

**Age range:** 14.00 Ma.

**Occurrence:** East Pacific, Ladera Formation, California, USA.

**Stratigraphic distribution:** Known from a single specimen found in the Ladera Formation, dated at 14.00 Ma based on the molluscan provincial stages (*Inuzuka, 2005*; *Domning & Barnes, 2007*).

## ***Neoparadoxia cecilialina Barnes, 2013***

**Age range:** 14.90-13.10 Ma.

**Occurrence:** East Pacific, Monterey Formation, California, USA.

**Stratigraphic distribution:** Known from a single specimen found in the Monterey Formation. *Barnes (2013)* assigned the age 11.00-10.00 Ma based on diatom stratigraphy. However, *Parham et al. (2022)* performed reassessment of this Formation based on diatom stratigraphy and faunal analysis, estimating its age to be 14.90-13.10 Ma.

## **Akan specimens (*Paleoparadoxia* sp. and *Neoparadoxia* sp.)**

**Age range:** 15.90-14.90 Ma.

**Occurrence:** West Pacific, Tonokita Formation, Hokkaido, Japan.

**Stratigraphic distribution:** Akan specimen found from one of the largest *Paleoparadoxia* fossil assemblage within the Tonokita Formation. *Urabe et al. (2003)* estimate the age of this Formation to be 15.90-14.90 Ma based on the diatom stratigraphy.

## ***Ashoroa laticosta Inuzuka, 2000***

**Age range:** 29.20-27.40 Ma.

**Occurrence:** West Pacific, Morawan Formation, Hokkaido, Japan.

**Stratigraphic distribution:** Known only from lower hared shale of the Morawan Formation. The age is based on fission-track (FT) dating and potassium-argon (K-Ar) dating (*Matsui & Ganzawa, 1987; Saito et al., 1988*), which suggest a late Oligocene age of 29.2 ± 3.3 Ma (FT)-27.4 ± 1.5 Ma (K-Ar).

## ***Cornwallius sookensis Cornwall, 1922***

**Age range:** 30.60-23.00 Ma.

**Occurrence:** North Pacific, Unalaska Formation, Unalaska Island, USA; East Pacific, Sook Formation, Vancouver Island, Canada; Yaquina Formation, Oregon, USA; San Gregorio Formation, Baja California Sur, Mexico and El Cien Formation, Baja California Sur, Mexico.

**Stratigraphic distribution:** The oldest record of *C. sookensis* comes from the Yaquina Formation, which *Prothero et al. (2001b)* estimated to date to 30.60-28.00 Ma based on magnetostratigraphy. The youngest fossil records are from the San Gregorio and El Cien formations, which *Kracht & Ganz (1998)* dated to 28.40-23.00 Ma.

## ***Ounalashkastylus tomidai Chiba et al., 2016***

**Age range:** 23.03 Ma.

**Occurrence:** North Pacific, Unalaska Formation, Unalaska Island, USA.

**Stratigraphic distribution:** *Jacobs et al. (2007)* dated the Unalaska Formation as between 24.10-13.00 Ma. *Chiba et al. (2016)* noted the locality falls near the Oligocene-Miocene boundary (23.03 Ma), or slightly younger.

## ***Desmostylus japonicus Tokunaga & Iwasaki, 1914***

**Age range:** 18.00-17.00 Ma.

**Occurrence:** West Pacific, Yamanouchi Formation, Gifu, Japan.

**Stratigraphic distribution:** The holotype of *D. japonicus* known from Yamanouchi Formation was deposited between 18.00-17.00 Ma based on diatomstratigraphy (*Kohno, 2000*). *Inuzuka et al. (1994)* assigned several specimens assigned *D. japonicus*, but did not provide any justification (*Matsui & Pyenson, 2023*). Therefore, our study follows only to the age of the holotype locality.

## ***Desmostylus hesperus Marsh, 1888***

**Age range:** 23.00-8.50 Ma.

**Occurrence:** Fossil occurrences of *D. hesperus* range from the USA, Mexico, Russia and Japan (*e.g., Reinhart, 1959; Ijiri & Kamei, 1961*).

**Stratigraphic distribution:** The oldest record of *D.hesperus* comes from Painted Sandstone Member of the ‘Vaqueros’ Formation assigned Aquitanian (23.00-20.40 Ma; *Mitchell & Repenning, 1963*). *Santos et al. (2016)* described the youngest record of *Desmostylus* from the Puente Formation, dated to 13.70-8.50 Ma (*Perham et al., 2022*). This fossil record suggests that *Desmostylus* may have existed until the late Miocene. Therefore, our study incorporates the record from the Puente Formation.

## ***Desmostylus (Vanderhoofius) coalingensis Reinhart, 1959***

**Age range:** 16.00-11.61 Ma.

**Occurrence:** East Pacific, Temblor Formation, California, USA.

**Stratigraphic distribution:** The classification of *D. coalingensis* is still debated (*e.g.,Inuzuka, 1994*; *Kohno, 2000*; *Santos et al., 2016*), and our study follows only to the age of the holotype locality. The holotype comes from Temblor Formation assigned 16.00-11.61 Ma (*Reinhart, 1959*).

# **Reference**

Barboza MM, Parham JF, Santos G, Kussman BN, Velez-Juarbe J. 2017. The age of the Oso Member, Capistrano Formation, and a review of fossil crocodylians from California. *PaleoBios* **34**:1-16 DOI [10.5070/P9341033797](https://doi.org/10.5070/P9341033797).

Barnes LG. 2013. A new genus and species of Late Miocene paleoparadoxiid (Mammalia, Desmostylia) from California. *Contributions in Science* **521**:51-114.

Beatty BL, Cockburn TC. 2015. New insights on the most primitive desmostylian from a partial skeleton of *Behemotops* (Desmostylia, Mammalia) from Vancouver Island, British Columbia. *Journal of Vertebrate Paleontology* **35**:e979939

DOI 10.1080/02724634.2015.979939.

Brandon MT, Roden-Tice MK, Garver JI. 1998. Late Cenozoic exhumation of the Cascadia accretionary wedge in the Olympic Mountains, northwest Washington State. *Geological Society of America Bulletin* 110:985–1009.

DOI [10.1130/0016-7606(1998)110<0985:LCEOTC>2.3.CO;2](https://doi.org/10.1130/0016-7606(1998)110%3C0985:LCEOTC%3E2.3.CO;2).

Chiba K, Fiorillo AR, Jacobs LL, Kimura Y, Kobayashi Y, Kohno N, Nishida Y, Michael P J, Tanaka K. 2016. A new desmostylian mammal from Unalaska (USA) and the robust Sanjussen jaw from Hokkaido (Japan), with comments on feeding in derived desmostylids. *Historical Biology* **28**:289-303

DOI 10.1080/08912963.2015.1046718.

Clark JM. 1991. A new early Miocene species of *Paleoparadoxia* (Mammalia: Desmostylia) from California. *Journal of Vertebrate Paleontology* **11**:490-508

DOI 10.1080/02724634.1991.10011417.

Cornwall IE. 1922. Some Notes on the Sooke Formation, Vancouver Island, B.C. T*he*

*Canadian Field-Naturalist* **36**:121–123 DOI [10.5962/p.338167](https://doi.org/10.5962/p.338167).

Domning DP, Barnes LG. 2007. A new name for the Stanford Skeleton of *Paleopara*

*doxia* (Mammalia, Desmostylia). *Journal of Vertebrate Paleontology* **27**:748-751 DOI 10.1671/0272-4634(2007)27[748:ANNFTS]2.0.CO;2.

Domning DP, Ray CE, McKenna MC. 1986. Two new oligocene desmostylians and a discussion of tethytherian systematics. *Smithsonian Contributions to Paleobiology* **59**:1-56 DOI 10.5479/si.00810266.59.1.

Ijiri S, Kamei T. 1961. On the skulls of *Desmostylus mirabilis* Nagao from South

Sakhalin and of *Paleoparadoxia tabatai* (Tokunaga) from Gifu Prefecture, Japan. *Earth Science* **53**:1-27 DOI [10.15080/agcjchikyukagaku.1961.53_1](http://dx.doi.org/10.15080/agcjchikyukagaku.1961.53_1).

Inuzuka N. 2000. Primitive Late Oligocene Desmostylians from Japan and Phylogeny

of the Desmostylia. *Bulletin of the Ashoro Museum of Paleontology* **1**:91-124.

Inuzuka N. 2005. The Stanford skeleton of *Paleoparadoxia* (Mammalia: Desmostylia).

*Bulletin of the Ashoro Museum of Paleontology* **3**:3-110.

Inuzuka N, Domning DP, Ray CE. 1994. Summary of taxa and morphological

adaptations of the Desmostylia. *Island Arc* **3**:522–537

DOI [10.1111/j.1440-1738.1994.tb00131.x](https://doi.org/10.1111/j.1440-1738.1994.tb00131.x).

Jacobs LL, Fiorillo AR, Gangloff R, Pasch A. 2007. Desmostylian remains from

Unalaska Island, Aleutian Chain, Alaska. *Bulletin of Carnegie Museum of Natural History* **39**:189–202 DOI 10.2992/0145-9058 (2007)39[189:DRFUIA]2.0.CO;2.

Kohno N. 2000. A centenary of studies on the holotype (NSM-PV 5600) of *Desmostylus japonicus* Tokunaga and Iwasaki, 1914. Bulletin of Ashoro Museum of Paleontology **1**:137-151.

Kracht O, Ganz B. 1998. Geochemistry and formation of phosphorites in the upper Oligocene San Gregorio Formation at La Purisima, Baja California Sur, Mexico. *Zentralblatt der Geologie und Paläontologie, Teil* **1**:471–483.

Matsui K, Kawabe S. 2015. The oldest record of Paleoparadoxia from the Northwest Pacific with an implication on the early evolution of Paleoparadoxiinae (Mammalia: Desmostylia). *Paleontological Research* **19**:251-265

DOI 10.2517/2015PR007.

Matsui K, Pyenson ND. 2023 New evidence for the antiquity of *Desmostylus* (Desmostylia) from the Skooner Gulch Formation of California. *Royal Society open science* **10**:221648 DOI 10.1098/rsos.221648.

Matsui K, Tsuihiji T. 2019. The phylogeny of desmostylians revisited: proposal of new clades based on robust phylogenetic hypotheses. *PeerJ* **7**:e7430

[DOI 10.7717/peerj.7430](http://doi.org/10.7717/peerj.7430).

Matsui M, Ganzawa Y. 1987. Oligo-Miocene Kawakami Group in Eastern Hokkaido. The age and horizon of Ashoro fossil fauna. *The Professor Masaru Matsui Memorial Volume* **137**:43.

Marsh OC. 1888. Notice of a new fossil sirenian from California. *American Journal of Science* **35**:94–96 DOI [10.2475/​ajs.s3-35.205.94](https://doi.org/10.2475/ajs.s3-35.205.94).

Mitchell ED, Repenning CA. 1963. The chronologic and geographic range of desmostylians. *Contributions in science* **78**:1–20. DOI 10. 5962/p.241026.

Parham JF, Barron JA. Velez-juarbe J. 2022. Middle and late Miocene marine mammal

assemblages fromthe Monterey Formation of Orange County, California. *The Geological Society of America Special Paper* **556**:1–14

DOI 10.1130/2021.2556(10).

Phillips FJ, Welton BJ, Welton J. 1976. Paleontologic studies of the middle Tertiary Skooner Gulch and Gallaway Formations at Point Arena, California. In *Meeting Program and Abstracts of the The Neogene symposium, Society of Economic Paleontologists and Mineralogist Annual Meeting, San Francisco, United States*, 137–154.

Prothero DR, Streig A, Burns C. 2001a. Magnetic stratigraphy and tectonic rotation of the Upper Oligocene Pysht Formation, Clallam County, Washington. *Pacific Section SEPM Book* **91**:224- 233.

Prothero DR, Bitboul CZ, Moore GW, Niem AR. 2001b. Magnetic stratigraphy and tectonic rotation of the Oligocene Alsea, Yaquina, and Nye formations, Lincoln County, Oregon. *Pacific Section SEPM Special Publication* **91**:184–194.

Prothero DR, Draus E, Cockburn TC, Nesbitt EA. 2008. Paleomagnetism and

counterclockwise tectonic rotation of the Upper Oligocene Sooke Formation,

southern Vancouver Island, British Columbia. *Canadian Journal of Earth Sciences* **45**:499–507 DOI [10.1139/E08-012](https://doi.org/10.1139/E08-012).

Reinhart RH. 1959. A review of the Sirenia and Desmostylia. *University of California Publications in Geological Sciences* **36**:1-146.

Saito T, Barron JA, Sakamoto M. 1988. An early Late Oligocene age indicated by diatoms for a primitive desmostylian mammal *Behemotops* from eastern Hokkaido, Japan. *Proceedings of the Japan Academy* **64B(9)**:269-273.

Santos GP, Parham JF, Beatty BL. 2016. New data on the ontogeny and senescence of

*Desmostylus* (Desmostylia, Mammalia). *Journal of Vertebrate Paleontology* **36**:2

DOI 10.1080/02724634.2016.1078344.

Shipps BK, Mauricio PC, Pyenson ND. 2019. *Borealodon osedax,* a new stem mysticete

(Mammalia, Cetacea) from the Oligocene of Washington State and its implications for fossil whale-fall communities. *Royal Society Open Science* **6**: 182168

DOI [10.1098/rsos.182168](https://doi.org/10.1098/rsos.182168).

Tokunaga S, Iwasaki C. 1914. Notes on *Desmostylus japonicus*. *Journal of the Geological Society of Tokyo* **21(255)**;33–33 DOI 10.5575/geosoc.21.255_33.

Tokunaga S. 1939. A new fossil mammal belonging to the Desmostylidae. . *In, Jubilee*

*Publication in Commemorating Professor H. Yabe, M.I.A., Sixtieth Birthday* **1**:289–299 Institute of Geology and Paleontology, Tohoku Imperial University, Sendai.

Urabe A, Akiha F, Hoyanagi K. 2003. Diatom biostratigraphy of the Neogene strata in

the Akan area, the eastern Hokkaido, northern Japan. *Journal of the Geological Society of Japan* **109(7)**:399-413  DOI [10.5575/geosoc.109.399](https://doi.org/10.5575/geosoc.109.399).
